# Profiles of Extrapulmonary Nontuberculous Mycobacteria Infections and Predictors for Species: A Multicenter Retrospective Study

**DOI:** 10.3390/pathogens9110949

**Published:** 2020-11-14

**Authors:** Jung Ho Kim, In Young Jung, Je Eun Song, Eun Jin Kim, Jun Hyoung Kim, Woon Ji Lee, Hye Seong, Jin Young Ahn, Su Jin Jeong, Nam Su Ku, Jun Yong Choi, Joon-Sup Yeom, Young Goo Song

**Affiliations:** 1 Department of Internal Medicine, Yonsei University College of Medicine, Seoul 03722, Korea; qetu1111@yuhs.ac (J.H.K.); junhy138@yuhs.ac (J.H.K.); leewj86@yuhs.ac (W.J.L.); shininghye@yuhs.ac (H.S.); jsj@yuhs.ac (S.J.J.); smileboy9@yuhs.ac (N.S.K.); seran@yuhs.ac (J.Y.C.); joonsup.yeom@yuhs.ac (J.-S.Y.); imfell@yuhs.ac (Y.G.S.); 2 Department of Internal Medicine, Yonsei University Wonju College of Medicine, Wonju 26426, Korea; vbsass@naver.com; 3 Department of Internal Medicine, Inje University College of Medicine, Goyang 10380, Korea; girasol531@naver.com; 4 Department of Internal Medicine, Ajou University School of Medicine, Suwon 16499, Korea; stone0128@ajou.ac.kr

**Keywords:** nontuberculous mycobacteria, rapidly growing mycobacteria, slowly growing mycobacteria

## Abstract

Extrapulmonary nontuberculous mycobacteria (NTM) infections contribute to morbidity and mortality worldwide. However, studies about extrapulmonary NTM infections have been limited. Therefore, we aim to describe the diversity of extrapulmonary NTM infections and identify predictors for species. Information regarding diversity of NTM isolates, antimicrobial susceptibility testing, treatment regimens, and outcomes were collected from four tertiary care centers in South Korea. Comparisons were made between patients with rapidly growing mycobacteria (RGM) and slowly growing mycobacteria (SGM) infections. A total of 117 patients (46 males vs. 71 females) were included. Skin and soft tissue infections (SSTIs) predominated (34.2%), followed by bone and joint infections (28.2%). In SSTIs, RGM species were predominantly identified (26/28, 92.9%), whereas SGM species were mainly identified in bone and joint infections (18/26, 69.2%), and the difference of isolated sites was verified by a post hoc test (*p* < 0.001). Multivariable regression analysis revealed that male sex (vs. female sex; OR 5.30, CI 1.35–24.26, *p* = 0.020) and bone and joint infections (vs. SSTIs; OR 18.10, CI 3.28–157.07, *p* = 0.002) were predictors of SGM infections, whereas the opposite was observed for RGM infections. Bone and joint infections and male sex were predictors for SGM infections, whereas SSTIs and female sex were predictors for RGM infections.

## 1. Introduction

Nontuberculous mycobacteria (NTM) are ubiquitous environmental organisms most commonly found in water and soil, and are a cause of pulmonary and extrapulmonary infections [1]. NTM disease is on the rise worldwide and is an important cause of morbidity and mortality [2,3]. Advances in identification techniques have increased physicians’ awareness of the heterogeneity of NTM isolates, however the surge in the number of cases cannot be solely explained by those advances and increased awareness [4].

NTM can be categorized into slowly growing mycobacteria (SGM) and rapidly growing mycobacteria (RGM), according to Runyon’s classification [5]. Species comprising SGM such as *Mycobacterium avium* complex are the most common NTM species responsible for diseases. However, infections caused by RGM such as *M. abscessus* complex are more difficult to treat due to antimicrobial drug resistance [6,7].

Extrapulmonary NTM infections include skin and soft tissue, bone and joint, urinary tract and lymph node infections. They are typically sporadic, but may be associated with clinical procedures or nosocomial outbreaks [8,9]. Recent retrospective studies of laboratory records from several countries have found that 20% to 30% of NTM isolates are of extrapulmonary origin [10,11,12]. However, studies on extrapulmonary NTM infection have been limited to case reports or population-based epidemiological studies due to their rarity; as a result, there is a lack of information on the microbiology, antimicrobial susceptibility testing (AST), and treatment regimens [13,14,15].

In NTM infection, the choice of antimicrobial regimens depends on whether the isolated NTM species are SGM or RGM. Conventional methods of specifying NTM isolates were based upon growth characteristics on solid media and subsequent biochemical tests, requiring additional weeks for subcultures [5]. Recent developments in molecular techniques have accelerated NTM isolation; however, cost matters limit the widespread adoption of these methods [16]. Therefore, in NTM infections, it is sometimes occurred to start empirical antimicrobial treatment before species identification. In particular, for bone and joint infections, repeated culture might be difficult due to invasiveness, and treatment may need to be started with limited information. In this case, selecting antimicrobial regimens could be determined depending on whether the isolated NTM species have a high probability of SGM or RGM; however, studies on predictors of SGM or RGM have lacked.

In addition, considering the geographic and ethnic variations of NTM infections, there is also a lack of research on extrapulmonary NTM infection found in Asia [17,18]. Species-specific and region-specific data that integrate clinical and microbiology information are crucial in determining treatment plans. Therefore, we performed a multicenter retrospective study to describe the diversity of NTM infections and correlate these observations with clinical information to examine the factors associated with disease caused by SGM or RGM species.

## 2. Results

### 2.1. Baseline Characteristics

**Figure 1:**
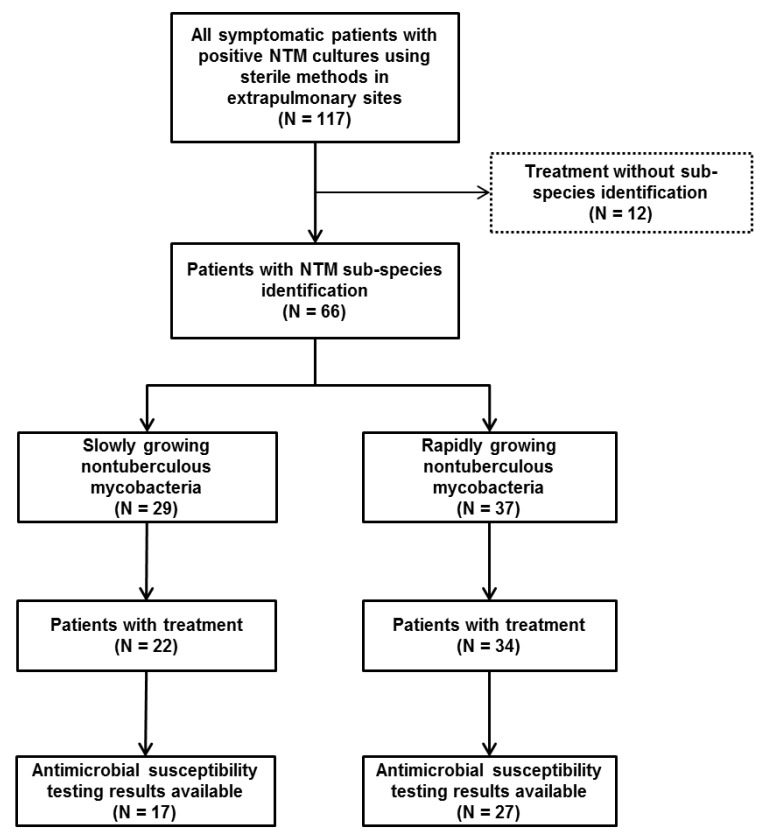
Study population and study flow. NTM: nontuberculous mycobacteria.

There were 117 patients that met the patient criteria defined in this study (Figure 1). Among them, 71 patients were female (60.7%), and the mean age was 53.1 ± 15.6 years. Twenty-four patients (20.5%) had diabetes mellitus, 16 patients (13.7%) received immunosuppressive treatment, and only one patient had HIV infection. NTM infections associated with surgery were most common (17.1%), followed by those associated with local injection (14.5%), which mainly included cosmetic procedures (7/17 cases) and injections for pain control (7/17 cases). Additional information including laboratory findings is shown in Table 1.

### 2.2. Diversity and Sample Sites of NTM Infections

**Figure 2:**
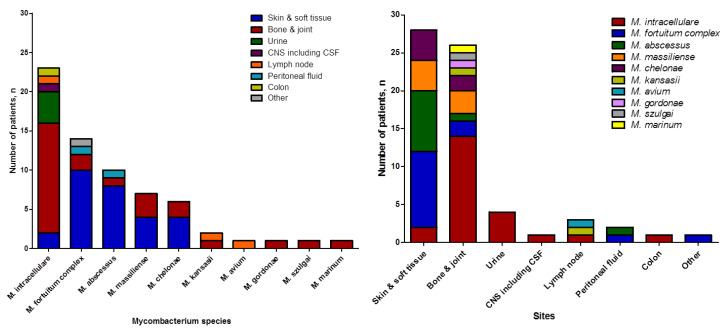
Species and site of infection of nontuberculous mycobacteria isolates. CNS: central nervous system; CSF: cerebrospinal fluid.

Figure 2 shows the species of NTM and their isolated sites. Skin and soft tissue infection was the most common extrapulmonary NTM infection (34.2%), followed by bone and joint infection (28.2%). *M. intracellulare* (34.8%) was the most common species identified, followed by RGM species such as *M. fortuitum* complex (21.2%), *M. abscessus* subsp. *abscessus* (15.2%), *M. abscessus* subsp. *massiliense* (10.6%) and, *M. chelonae* (9.1%). RGM species were predominantly identified (26/28, 92.9%) in skin and soft tissue infections, while SGM species were mainly identified in bone and joint infections (18/26, 69.2%).

### 2.3. Antimicrobial Susceptibility Testing of NTM Infections

The AST results of NTM isolates varied depending on whether SGM or RGM were cultured. *M. intracellulare* were all susceptible to clarithromycin (100%), but they were rarely susceptible to moxifloxacin (13.3%) and were not susceptible at all to linezolid (0%). In the case of RGM, AST results were varied according to species. Clarithromycin showed good activity against both *M. abscessus* subsp. *massiliense* and *M. chelonae* (susceptibility rate 85.7% and 80%, respectively), but not against *M. fortuitum* complex and *M. abscessus* subsp. *abscessus* (susceptibility rate 50% and 25%, respectively). Most of RGM were susceptible to amikacin and linezolid also showed good activity against RGM species including *M. abscessus* subsp. *abscessus* (susceptibility rate 100%). *M. fortuitum* complexes were all susceptible to fluoroquinolone, but *M. abscessus* subsp. *abscessus* was not (Table 2).

### 2.4. Antimicrobial Regimens in Patients with NTM Infections

Antimicrobial regimens chosen for patients with NTM infections tended to follow American Thoracic Society recommendations in the case of SGM infections [5]. For example, treatment of *M. intracellulare* mainly comprised clarithromycin, rifampicin, and ethambutol. The median duration of medical treatment was 12 months. In the case of RGM infections, fluoroquinolone and clarithromycin were mainly used as treatment, whereas amikacin, cefoxitin, and imipenem, which are injection antimicrobials, were not used often. More information about the median duration of treatment, the average number of antimicrobials used, and which antimicrobials were used are depicted in Table 3.

### 2.5. Comparison of Clinical Characteristics and Predictors between SGM and RGM Infections

Table 4 shows a comparison of clinical characteristics between SGM and RGM infections. Among the 66 patients whose NTM species were identified, 29 were infected with SGM and 37 were infected with RGM. The proportion of females was higher in RGM-infected cases than in SGM-infected cases (81.1% vs. 37.9%; *p* < 0.001). In skin and soft tissue infections, RGM species were predominantly identified (26/28, 92.9%), whereas SGM species were mainly identified in bone and joint infections (18/26, 69.2%). The difference between the isolated sites was verified by a post-hoc test (*p* < 0.001). There were no significant differences between the two groups in terms of comorbidities, predisposing factors of infection, laboratory findings, patients who had received surgical treatment, duration of medical treatment, and treatment outcomes. Multivariable logistic regression analysis revealed that the male sex (vs. female sex; odds ratio (OR) 5.30, 95% confidence interval (CI) 1.35–24.26; *p* = 0.020) and bone and joint infections (vs. skin and soft tissue infections; OR 18.10; 95% CI 3.28–157.07; *p* = 0.002) were predictors of SGM infections, whereas the opposite was observed for RGM infections. These results were found to be robust after checking the interaction between sex and isolated sites (*p* > 0.05). The results of multivariate logistic regression analysis are shown in Table 5.

## 3. Discussion

In this multicenter retrospective study, we described the clinical characteristics of patients with extrapulmonary NTM infections. The differences in isolated sites, AST results, and the antimicrobial regimens chosen according to the NTM species were also described. We then compared SGM and RGM infections; the results showed differences between the two groups depending on the sex and isolated sites.

SGM and RGM showed significant differences in their isolated sites. Skin and soft tissue infections were predominantly caused by RGM, while bone and joint infections were mainly caused by SGM. It is well known that skin and soft tissue NTM infection is frequently associated with local injection and mainly caused by RGM [19]. However, there is a lack of information on the common causative species of bone and joint NTM infections. In our study, *M. intracellulare* was identified mainly in bone and joint infections, and this result is consistent with the results of another study [20]. Since *M. intracellulare* was identified in all of the bone and joint NTM infections after orthopedic surgery, orthopedic surgery could be the cause of bone and joint infection caused by *M. intracellulare*; however, no statistically significant evidence was provided due to the small number of cases.

There has been no consistent result regarding the differences in extrapulmonary NTM infections according to sex. In a French hospital, the proportion of females with extrapulmonary NTM infection was 34.6% (27/78), while in the US, 52% (70/134) of the extrapulmonary NTM-infected patients were female [21,22]. In our study, 60.7% of NTM-infected patients were female, which was mainly due to the high rate of female patients with RGM infections (81.1%). The high incidence rate of female patients with extrapulmonary NTM infections, particularly RGM infections, is consistent with the results of studies conducted in Taiwan and South Korea. In Taiwan, 73.3% (22/30) of patients with SSTIs due to RGM were female, and in the study conducted in South Korea, 70% (14/20) of patients with *M. abscessus* complex infections were female [23,24]. One of the reasons for the high rate of female patients with RGM infections might be due to the fact that the proportion of females receiving local injection such as cosmetic procedures, one of the predisposing factors for extrapulmonary NTM infections, was higher than that for males (88.2% vs. 11.8%; *p* = 0.014). Meanwhile, previous studies reported that the anti-IFNγ autoantibody is more common in Asians, and it has been suggested that this may increase susceptibility to extrapulmonary NTM infections, but the genetic predisposition by sex was not identified [25,26]. The effect of interaction between sex and isolated sites was not statistically significant.

Despite the effectiveness of the usual antimicrobial regimens according to the guidelines, it is still necessary to upgrade the knowledge concerning the treatment of extrapulmonary NTM infections. The results of AST were similar to those previously reported, but there were some notable differences. First, the results of our study that SGM were susceptible to clarithromycin and most RGM were susceptible to amikacin, together with the predictors of SGM and RGM revealed in this study, might be helpful in early empirical antimicrobial regimen selection. On the other hand, *M. abscessus* subsp. *abscessus* is generally known to be resistant to linezolid, but in this study, all identified *M. abscessus* subsp. *abscessus* isolates were susceptible [27]. Therefore, clinicians should pay more attention to AST in NTM infections since the commonly known AST results do not necessarily correspond to confirmed AST results. Even if treatment with an initial antimicrobial agent is initiated according to the existing recommendations, the antimicrobial regimen should be tailored thereafter by combining knowledge of AST results and clinical response.

The regimens and duration of antimicrobial treatment for extrapulmonary NTM infections remain poorly established [28,29]. Treatment regimens were suggested to be based on in vitro susceptibility test results. For *M. intracellulare* infections, a macrolide-based regimen was used in most patients because all patients with *M. intracellulare* infections were susceptible to clarithromycin. In the case of RGM infections, amikacin and other injection antimicrobials were not commonly used. This was mainly due to the fact that RGM infections were predominantly identified in skin and soft tissue, and were combined with effective surgical treatment, the prognosis of RGM infections appeared to be good even if no injection antimicrobial was used. This also means that surgical treatment should be considered in the case of extensive disease and abscess formation, as well as when drug therapy is difficult [5].

This study had some limitations. First, it was a nonrandomized retrospective study, and different individuals at each institution recorded the information included in the study. However, we tried to minimize bias by using a standardized case report form. Second, this study was conducted in a single country with a homogenous ethnic background. Thus, the results of this study might be different from studies conducted in other countries in terms of AST results. Third, NTM species from 51 patients remained unidentified. In order to identify the species of NTM in South Korea, it is necessary to request identification of the isolates from outside referral institutions. Clinicians who are not familiar with the process may often begin treatment without species identification. In order to better characterize comorbidities and predisposing factors, we included not only patients with their NTM species identified, but also patients who were clinically diagnosed NTM infection based on culture results obtained through sterile methods. To minimize any misunderstanding, in the isolated sites, AST, and antimicrobial regimens, we described the cases separately for those with species identified and those in which species were not identified. Finally, although this was a multicenter study, the number of patients included, especially the number of patients with species identification was not large. Therefore, there was a limit to the comparison of the therapeutic effect of treatment duration and combination of antimicrobial regimens for extrapulmonary NTM infections. However, in a situation where the information on susceptibility patterns and treatments of NTM infections is very limited, we believe that the information on susceptibility patterns of each species and therapeutic antimicrobial regimens as well as treatment duration can provide useful information for the treatment of extrapulmonary NTM infections. Subsequent multinational and multicenter studies would be beneficial in this regard.

In conclusion, bone and joint infections and male sex were predictors for SGM infections, whereas skin and soft tissue infections and female sex were predictors for RGM infections. AST results and treatment regimens of NTM isolates varied depending on whether the infection was caused by SGM or RGM. Since species identification is fastidious and takes time in NTM infections, identifying predictors of SGM and RGM can be of great help to treat patients. Besides, the information of AST and selected antimicrobial regimens in this study could guide to choose appropriate early antimicrobial regimens, and therefore, can help to improve patient prognosis. Although this study does not represent a worldwide survey, it does provide a snapshot of extrapulmonary NTM infections that are prevalent in our environment. Clinicians should be aware of the species-specific microbiology information of extrapulmonary NTM infections.

## 4. Materials and Methods

### 4.1. Study Design and Case Definitions

We retrospectively analyzed data of all symptomatic patients with positive NTM cultures from extrapulmonary sites using sterile methods at four tertiary-care centers in South Korea between January 2006 and June 2018. Extrapulmonary sites include skin and soft tissue, bone and joint, urinary tract, and the central nervous system (CNS) including cerebrospinal fluid (CSF), lymph node, peritoneal fluid, colon tissues, and other sites with the exception of respiratory tracts. We included symptomatic patients with NTM isolated from sterile sites such as bone and joint, CSF, lymph node, and peritoneal fluid. In cases where NTM was isolated from nonsterile sites, criteria for defining a true case of NTM infection were applied as follows: exclusion of extrapulmonary tuberculosis; and medical record with a specific diagnosis of NTM infection made by a physician [30]. We also decided to include one patient with an isolate of *M. gordonae*, which is generally considered to be nonpathogenic; however, after reviewing medical records, it was concluded that the patient had true bone and joint infection with *M. gordonae*. We performed additional analysis for patients in whom species were identified at isolated sites such as AST and a comparison between SGM and RGM infections. We used a standardized case report form to collect patient information including demographics, comorbidities, predisposing factors, diversity of NTM isolates, AST, treatment regimens, and outcomes. This study was approved by the Institutional Review Board (IRB) of the Yonsei University Health System Clinical Trial Center (4-2019-0794). The IRB waived the requirement for informed consent from the patients, since the study was purely observational in nature, and the study subjects were anonymized.

### 4.2. Definitions of Variables

We defined skin and soft tissue infection as a case with one or more isolates from tissue samples labeled as, “furuncles,” “abscesses,” “skin,” “wound,” or “incisions” [11]. Bone and joint infection was defined as a case with one or more isolates from bone biopsy or joint fluid cultures [21]. Lymph node infection was defined as a case with isolation of NTM from a lymph node biopsy or aspirate [11]. The categories of “urine”, “CNS including CSF”, and “peritoneal fluid” infections were used in cases that had positive cultures at the respective sites along with relevant symptoms [11]. In particular, the category of “peritoneal fluid” infection included only cases of more than 250 polymorphonuclear cells per μL in peritoneal fluid. Colon infection was defined as a case with one or more isolates from tissue biopsy samples obtained by endoscopy along with relevant symptoms such as abdominal pain or diarrhea [31]. History of previous tuberculosis was defined as a case with self-reported history of physician-diagnosed tuberculosis or lesions detected on chest radiographs [32]. Immunosuppressive therapy was defined as any treatment that lowered the activity of the body’s immune system [33]. History of acupuncture was defined as a case with the development of extrapulmonary NTM infection in the area of invasive acupuncture treatment [34]. Local injection included subcutaneous, intraarticular, and periarticular injections [35]. Indwelling-catheter-related infection was defined as isolation of NTM from normally sterile material [21].

### 4.3. Specimen Processing and Antimicrobial Susceptibility Testing

Specimen processing was carried out according to the national guidelines in each hospital as follows [36]. Clinical specimens from nonsterile sites were decontaminated with 4% NaOH. All specimens were homogenized, and then concentrated by centrifugation at 3000 g for 18 min. Fluorochrome stain using auramine–rhodamine was applied to examine a portion of sediment. The concentrated specimens were cultured in 3% Ogawa medium and inspected on a weekly basis until eight weeks postinoculation. A portion of the specimens was mixed with pH 6.8 phosphate buffer solution. Then, 0.5 mL of the mixture was cultured in mycobacteria growth-indicator tube medium (Becton Dickinson, Franklin Lakes, NJ, USA) and observed until six weeks postinoculation [5,37]. For NTM species identification and AST, each hospital sent samples to Seoul Clinical Laboratories (SCL, Seoul, South Korea), and the samples were analyzed in the following manner: NTM species were identified using a line probe assay with MolecuTech REBA Myco-ID kit (YD diagnostics, Yongin, South Korea), which included reverse blot hybridization assay. For further species identification of the isolates, sequencing analysis of the 16S rRNA gene, 16S-23S rRNA internal transcribed spacer sequences, partial *hsp65* sequences, and partial *rpoB* sequences was performed [24]. AST was performed in accordance with the guidelines of the Korean Institute of Tuberculosis [38]. The minimum inhibitory concentration was defined as the lowest drug concentration required to inhibit visible growth of the mycobacteria. Susceptibility and resistance of NTM species to specific antimicrobials were evaluated using previously established standards [38].

### 4.4. Statistical Analysis

We described the baseline characteristics, infection sites, AST results, and treatment regimens of patients with NTM infections. Comparisons were made between patients with SGM and RGM infections. The Kolmogorov-Smirnov test and Shapiro-Wilk test were conducted to verify the normality of the distribution of continuous variables. To compare continuous variables, Student’s t-test and the Mann-Whitney U test were used, while chi-squared tests or Fisher’s exact tests were used to compare categorical variables. Post hoc testing was performed using the Bonferroni method to determine whether there were differences in the isolated sites according to species, and the predictive factors for SGM or RGM infections were determined by multivariable logistic regression analysis. The results were expressed as ORs and 95% CIs. *p* < 0.05 was considered statistically significant. All statistical analyses were performed using R studio Version 1.1.463-© 2009-2018 R studio, Inc.

## Figures and Tables

**Table 1:** Baseline Characteristics of 117 patients with nontuberculous mycobacteria infections.

Variables	N (%)
Age (years)	53.1 ± 15.6
Female sex, n (%)	71 (60.7)
**Comorbidities, n (%)**	
Diabetes mellitus	24 (20.5)
HIV infection	1 (0.9)
Solid organ cancer	10 (8.5)
Hematologic malignancy	4 (3.4)
History of previous tuberculosis	6 (5.1)
Chronic kidney disease	14 (12.0)
Chronic liver disease	4 (3.4)
Connective tissue disease	5 (4.3)
Immunosuppressive treatment	16 (13.7)
Antibiotics treatment within 30 days	40 (34.2)
**Predisposing factors, n (%)**	
Any surgery	20 (17.1)
Orthopedic Surgery	4 (3.4)
Plastic surgery	4 (3.4)
Other surgery	12 (10.3)
Local injection	17 (14.5)
History of acupuncture	6 (5.1)
Trauma	6 (5.1)
Indwelling catheter	4 (3.4)
Unknown	64 (54.7)
**Laboratory findings**	
ESR (mm/h)	33.0 (16.0–57.0)
CRP (mg/L)	11.55 (2.90–57.05)
IGRA positivity, n (%)	11/23 (47.8)

ESR: erythrocyte sedimentation rate; CRP: C-reactive protein; IGRA: interferon gamma release assay. Data are presented as mean ± standard deviation or median (interquartile range) or number (%) of patients.

**Table 2:** Antimicrobial susceptibility testing of nontuberculous mycobacteria isolates.

	Rapidly Growing Mycobacteria												Slowly Growing Mycobacteria								
	*M. fortuitum* Complex (N = 9)			*M. abscessus* subsp. *abscessus* (N = 6)			*M. abscessus* subsp. *massiliense* (N = 7)			*M. chelonae* (N = 5)			*M. intracellulare* (N = 15)			*M. kansasii* (N = 1)			*M. avium* (N = 1)		
	S	I	R	S	I	R	S	I	R	S	I	R	S	I	R	S	I	R	S	I	R
Amikacin	8	0	1	4	0	1	7	0	0	2	1	2									
Cefoxitin	2	6	0	2	2	0	6	1	0	0	0	5									
Ciprofloxacin	8	0	0	0	1	3	2	0	5	1	1	3									
Clarithromycin	4	1	3	1	0	3	6	0	1	4		1	15	0	0				1	0	0
Doxycycline	4	0	4	0	0	4	0	1	6	2	0	3									
Imipenem	6	2	0	2	1	0	5	2	0	0	4	1									
Moxifloxacin	9	0	0	0	1	3	2	2	3	0	1	4	2	4	9	1	0	0	0	1	0
TMPSMX	3	0	5	0	0	4	4	0	3	0	0	5									
Tobramycin										3	1	1									
Linezolid	6	2	0	3	0	0	6	0	1	4	0	1	0	8	7				0	0	1
Isoniazid	0	0	1	0	0	2										0	0	1			
Pyrazinamide	1	0	0	2	0	0										1	0	0			
Rifabutin	0	0	1	0	0	2										1	0	0			
Ethambutol	0	0	1	0	0	2										1	0	0			
Rifampicin	0	0	1	0	0	1										1	0	0			

S: Susceptible; I: Intermediate; R: Resistant; TMPSMX: Trimethoprim/sulfamethoxazole.

**Table 3:** Antimicrobial regimens chosen in patients with nontuberculous mycobacteria infections.

	Rapidly Growing Mycobacteria				Slowly Growing Mycobacteria			
	*M. fortuitum* complex	*M. abscessus* subsp. *abscessus*	*M. abscessus* subsp. *massiliense*	*M. chelonae*	*M. intracellulare*	*M. kansasii*	*M. gordonae*	*M. marinum*
No of patients *	13	8	7	6	18	2	1	1
Median treatment duration (months)	9.0	12.0	10.5	10.5	12.0	18.0	14.0	6.0
Mean numbers of antimicrobial used %	3.0	3.1	2.6	2.8	2.9	3.5	4.0	4.0
Amikacin	4	2	0	0	2	0	0	1
Cefoxitin	0	2	2	0	0	0	0	0
Ethambutol	4	1	1	1	13	2	1	0
Fluoroquinolone ^x^	10	5	4	4	7	0	1	1
Imipenem	1	2	1	1	1	0	0	0
Isoniazid	3	1	0	0	3	2	0	0
Linezolid	0	1	0	1	1	0	0	0
Macrolide ^‡^	8	8	7	6	15	1	1	1
Pyrazinamide	0	1	0	0	2	0	0	0
Rifamycin ^&^	5	2	1	1	16	2	1	0
Tetracycline ^#^	4	3	0	2	1	0	0	1
TMPSMX	3	0	2	0	1	0	0	0

* Indicates the number of patients for whom there is treatment regimen data available. ^x^ Fluoroquinolone includes ciprofloxacin (17 patients) or moxifloxacin (10 patients) or levofloxacin (10 patients). ^‡^ Macrolide includes azithromycin (five patients) or clarithromycin (51 patients). ^&^ Rifamycin includes rifampicin (29 patients) or rifabutin (three patients). ^#^ Tetracycline includes doxycycline (12 patients) or minocycline (three patients). TMPSMX: Trimethoprim/sulfamethoxazole.

**Table 4:** Comparison of clinical characteristics between slowly growing mycobacteria and rapidly growing mycobacteria.

	Univariate Analysis		
	Slowly Growing NTM (N = 29)	Rapidly Growing NTM (N = 37)	*p* Value
Age (years)	58.17 ± 15.36	51.84 ± 15.59	0.104
Female sex, n (%)	11 (37.9)	30 (81.1)	<0.001
**Isolated sites, n (%)**			<0.001 ^a^
Skin and soft tissue	2 (6.9)	26 (70.3)	
Bone and joint infection	18 (62.1)	8 (21.6)	
Others	9 (31.0)	3 (8.1)	
**Comorbidities, n (%)**			
Diabetes mellitus	6 (20.7)	11 (29.7)	0.580
Solid organ cancer	1 (3.4)	2 (5.4)	>0.999
Hematologic malignancy	1 (3.4)	2 (5.4)	>0.999
History of tuberculosis	3 (10.3)	2 (5.4)	0.647
Chronic kidney disease	2 (6.9)	4 (10.8)	0.688
Chronic liver disease	1 (3.4)	1 (2.7)	>0.999
Connective tissue disease	1 (3.4)	3 (8.1)	0.625
Immunosuppressive treatment	3 (10.3)	7 (18.9)	0.493
Antibiotics within 30 days	9 (31.0)	19 (51.4)	0.160
**Predisposing factors, n (%)**			0.480 ^a^
History of acupuncture	1 (3.4)	3 (8.1)	
Local injection	4 (13.8)	10 (27.0)	
Any surgery	5 (17.2)	9 (24.3)	
Orthopedic Surgery	3 (10.3)	1 (2.7)	
Plastic surgery	0 (0.0)	3 (8.1)	
Other surgery	2 (6.9)	5 (13.5)	
Trauma	2 (6.9)	2 (5.4)	
Indwelling catheter	0 (0.0)	2 (5.4)	
**Laboratory findings**			
ESR (mm/h)	41.0 (11.5–64.0)	32.0 (22–60)	0.692
CRP (mg/L)	10.0 (3.4–40.9)	12.4 (1.8–59.8)	0.913
IGRA positivity, n (%)	3/7 (42.9)	4/9 (44.4)	>0.999
**Treatment**			
Surgical treatment, n (%)	17 (58.6)	23 (62.2)	0.770
Duration of medical treatment (months)	12.0 (6.0–14.8)	9.0 (6.0–13.5)	0.683
**Treatment outcomes, n (%)**			
Cured	22 (75.9)	27 (73)	0.790
Died	1 (3.4)	2 (5.4)	1.000
Loss to follow up	6 (20.7)	8 (21.6)	0.927

^a^ Bonferroni adjusted p value via post hoc test. NTM: nontuberculous mycobacteria; ESR: erythrocyte sedimentation rate; CRP: C-reactive protein; IGRA: interferon gamma release assay. Data are presented as mean ± standard deviation or median (interquartile range) or number (%) of patients.

**Table 5:** Multivariate logistic regression analysis to identify predictive factors for slowly growing mycobacteria.

	Univariate Analysis		Multivariate Analysis	
	OR (95% CI)	*p* Value	OR (95% CI)	*p* Value
**Age**	0.97 (0.94, 1.00)	0.110	0.99 (0.94, 1.04)	0.700
**Sex**				
Female	1 (ref.)		1 (ref.)	
Male	7.01 (2.40, 22.63)	<0.001	5.30 (1.35, 24.26)	0.020
**Isolated sites**				
Skin and soft tissue	1 (ref.)		1 (ref.)	
Bone and joint	29.25 (6.65, 211.69)	<0.001	18.10 (3.28, 157.07)	0.002
Others	1.33 (0.30, 7.14)	0.720	2.13 (0.36, 16.67)	0.430

CI: confidence interval; OR: odds ratio.
